# Functional disability of adults in Brazil: prevalence and associated factors

**DOI:** 10.1590/S0034-8910.2015049005945

**Published:** 2015-12-16

**Authors:** Keitty Regina Cordeiro de Andrade, Marcus Tolentino Silva, Taís Freire Galvão, Maurício Gomes Pereira

**Affiliations:** I Faculdade de Medicina. Universidade de Brasília. Brasília, DF, Brasil; II Faculdade de Medicina. Universidade Federal do Amazonas. Manaus, AM, Brasil; III Hospital Universitário Getúlio Vargas. Universidade Federal do Amazonas. Manaus, AM, Brasil

**Keywords:** Adult, Mobility Limitation, Risk Factors, Statistics on Sequelae and Disability, Disabled Persons

## Abstract

**OBJECTIVE:**

To estimate the prevalence and factors associated with functional disability in adults in Brazil.

**METHODS:**

We used information from the health supplement of the National Household Sample Survey in 2008. The dependent variable was the functional disability among adults of 18 to 65 years, measured by the difficulty of walking about 100 meters; independent variables were: health plan membership, region of residence, state of domicile, education level, household income, economic activity, self-perception of health, hospitalization, chronic diseases, age group, sex, and color. We calculated the gross odds ratios (OR), and their respective confidence intervals (95%), and adjusted them for variables of study by ordinal logistic regression, following hierarchical model. Sample weights were considered in all calculations.

**RESULTS:**

We included 18,745 subjects, 74.0% of whom were women. More than a third of adults reported having functional disability. The disability was significantly higher among men (OR = 1.17; 95%CI 1.09;1.27), people from 35 to 49 years (OR = 1.30; 95%CI 1.17;1.45) and 50 to 65 years (OR = 1.38; 95%CI 1.24;1.54); economically inactive individuals (OR = 2.21; 95%CI 1.65;2.96); adults who reported heart disease (OR = 1.13; 95%CI 1.03;1.24), diabetes mellitus (OR = 1.16; 95%CI 1.05;1.29), arterial systemic hypertension (OR = 1.10; 95%CI 1.02;1.18), and arthritis/rheumatism (OR = 1.24; 95%CI 1.15;1.34); and participants who were admitted in the last 12 months (OR = 2.35; 95%CI 1.73;3.2).

**CONCLUSIONS:**

Functional disability is common among Brazilian adults. Hospitalization is the most strongly associated factor, followed by economic activity, and chronic diseases. Sex, age, education, and income are also associated. Results indicate specific targets for actions that address the main factors associated with functional disabilities and contribute to the projection of interventions for the improvement of the well-being and promotion of adults’ quality of life.

## INTRODUCTION

The functional disability is the difficulty or inability of performing basic daily activities within the normal standards of the human being.^a^ The major cause for this limitation is physical deficiency, which leads to impacts on the ability of developing social activities.^3,23^

According to the World Health Organization, about 10.0% of the population of developed countries comprises people with some kind of functional disability, this percentage rising to about 15.0% in developing countries.^b^

Functional disabilities are commonly measured by self-report.^3^ Daily life activities and physical mobility are often used for the assessment, being considered an important indicator of health.^13^

The international scientific community wants to understand the factors associated with this topic.^14^ However, we only observed a few population-based studies on the prevalence of functional disability among adults in the country. To know the distribution and to understand the factors that collaborate to functional disabilities may assist public policy planners in intervention projections for the improvement of the well-being and promoting the quality of life of adults.

The present study aimed to estimate the prevalence and the factors associated with functional incapacity of Brazilian adults.

## METHODS

We used information from the health supplement of the National Household Sample Survey (PNAD). It is a survey, carried out by the Brazilian Institute of Geography and Statistics, that obtained information from a probabilistic sample of 150,591 households and 391,868 individuals, from September 28 of 2007 to 27 of September of 2008.

The PNAD offers a complex sample design planned to allow the national representation obtained in three stages: (a) primary units – self-representative municipalities with probability of belonging to the sample, and not self-representatives, with probability of being part of the proportional sample of resident population; (b) secondary unities – census sectors, where the probability of inclusion is proportional to the number of existing homes in the sector; and (c) tertiary units – (private household and housing units in collective households), investigating the information related to all residents.^c^

This study included adults of between 18 and 65 years. Only people who have informed their own functional capacities were considered in the analysis, while *proxy *respondents were excluded.

The Health Supplement Survey of PNAD included seven questions on physical mobility regarding daily activities, sports, climbing stairs, and walking. Four ordinal answers were possible: “not able to do it”, “with great difficulty”, “with little difficulty” or “with no difficulty”.

The dependent variable was the functional disability measured by using the variable of physical mobility “difficulty to walk about 100 m” Independent variables were determined by blocks with distal to proximal components (Figure) to avoid the underestimation of the effects of distal variables:

**Figure:**
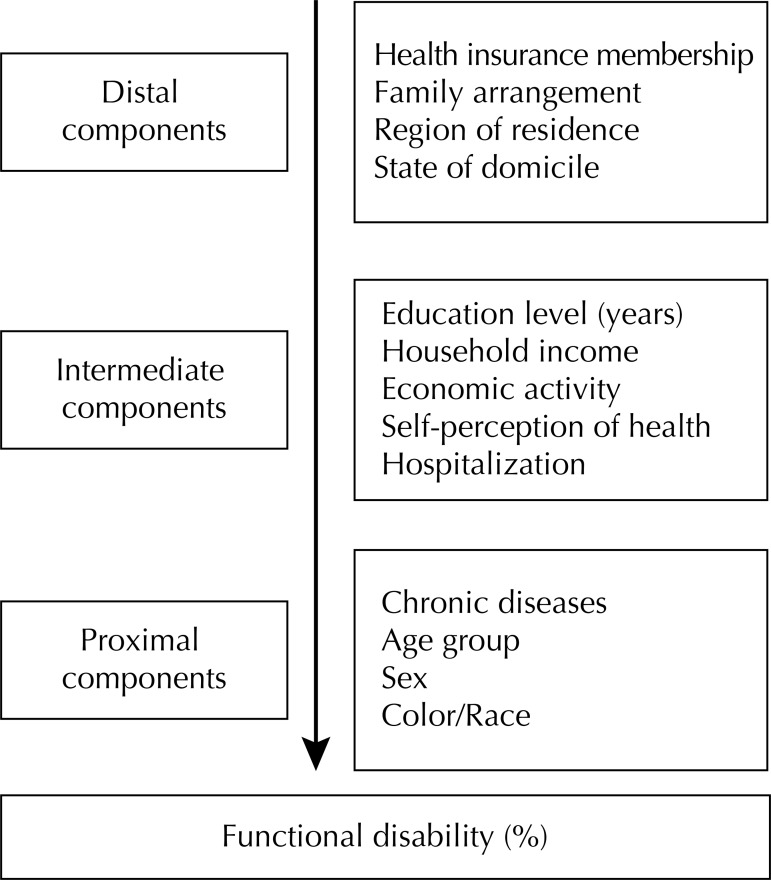
Graphic scheme of the hierarchical model used in the analysis. Brazil, 2015.

Block 1 distal components: health plan membership (yes; no), family arrangement (living alone; accompanied), region of residence (North, Northeast, Southeast, South, Midwest) and State of domicile (rural; urban).

Block 2 intermediate components: education level (zero to three years; four to seven years, eight to 11 years; and 12 or more years of study), *per capita* household income in tertiles (3^rd^ > R$507.00; 2^nd^, R$277.00 to R$507.00; and 1^st^ < R$276.00), economic activity (active; inactive), self-perception of health (good; moderate; bad) and hospitalization (in the last 12 months).

Block 3 proximal components: chronic diseases (back problems, arthritis/rheumatism, cancer, diabetes mellitus, bronchitis or asthma, systemic hypertension, heart diseases, renal insufficiency, depression, tendonitis), age group (years), sex (male; female), and color (white; non-white).

We obtained the descriptive statistics of variables stratified by functional disability. The calculation of self-referred prevalence was carried out in the total population with a respective 95% confidence interval (95%CI). To identify factors associated with functional incapacity, we used the bivariate analysis by adopting as effect measure the odds ratio (OR).

Odds ratios set were calculated using the model of ordinal logistic regression^1^ that came from three scenarios: (i) with difficulty *versus* (with little difficulty + with great difficulty + not able to do it); (ii) (with no difficulty + with little difficulty) *versus* (with great difficulty + not able to do it); and (iii) (with no difficulty + with little difficulty + with great difficulty) *versus* not able to do it. Such care was necessary because of the lack of mathematical linearity between categories under analysis.

The multivariate analysis was ranked by previously defined blocks. For each block of analysis, variables with values of p < 0,10 were kept in the model. Variables were adjusted by covariates of the same level and by significant variables of the previous level. The *Jackknife* technique was used for the sensitivity analysis, obtaining stratified simulations by the Federation Unit.

All analyses were conducted using the Stata statistical software version 10.1. Sample weights of PNAD were considered in all calculations.

The PNAD was approved by the National Committee of Ethics in Research.

## RESULTS

In total, we included 18,745 interviews in the study. Population was predominantly female and most adults had between 50 and 65 years, were living accompanied in an urban area, considered themselves as not-white, had up to seven years of study, belonged to the lowest income tertile and was economically inactive (Table 1).

**Table 1:** Distribution of adult’s characteristics and their associations with functional disabilities. Brazil, 2015. (N = 18,745)

Variables	Adults (%)	Functional disability (%)				Bivariate analysis		

		With no difficulty	With little difficulty	With great difficulty	Not able to do it	OR	95%CI	p
Health insurance membership								
Yes	20.2	67.2	24.9	6.6	1.3	0.81	0.75;0.89	< 0.001
Family arrangement								
Living alone	7.3	60.8	32.0	6.4	0.7	1.08	0.98;1.21	0.203
Region of residence								
North	8.3	64.6	28.9	6.0	0.6	1.00	–	–
Northeast	28.0	66.2	26.4	6.3	1.2	0.95	0.86;1.06	0.371
Southeast	40.0	61.7	29.1	7.8	1.4	1.16	1.05;1.29	0.005
South	16.7	61.4	30.0	7.6	1.0	1.16	1.04;1.30	0.010
Midwest	7.0	63.4	27.6	7.2	1.8	1.09	0.96;1.24	0.181
State of domicile								
Urban	82.4	62.5	29.0	7.3	1.3	1.22	1.12;1.33	< 0.001
Education level (years)								
≥ 12	21.0	70.0	24.2	4.6	1.2	1.00	–	–
8 to 11	16.6	66.4	25.3	6.9	1.4	1.21	1.08;1.35	0.001
4 to 7	33.9	61.7	29.9	7.4	1.0	1.45	1.32;1.59	< 0.001
0 to 3	28.4	58.4	31.4	8.8	1.5	1.68	1.53;1.85	< 0.001
*Per capita* household income^a^								
3^rd^ (> 507)	30.0	65.3	27.0	6.5	1.2	1.00	–	–
2^nd^ (277-507)	28.6	61.2	29.7	7.6	1.5	1.19	1.09;1.30	< 0.001
1^st^ (≤ 276)	41.4	63.2	28.4	7.3	1.1	1.09	1.01;1.18	0.027
Economic activity								
Inactive	43.4	56.7	32.0	9.5	1.8	1.68	1.58;1.79	< 0.001
Self-perception of health								
Good	31.0	73.0	22.2	3.8	1.1	1.00	–	–
Moderate	50.3	62.9	29.4	6.6	1.1	1.58	1.47;1.71	< 0.001
Bad	18.7	48.2	35.7	14.2	2.0	3.03	2.76;3.34	< 0.001
Hospitalization								
In the last 12 months	16.0	53.1	32.8	11.6	2.5	1.73	1.59;1.88	< 0.001
Chronic diseases								
Back problems	46.0	60.7	29.9	8.4	1.2	1.24	1.16;1.32	< 0.001
Arthritis or rheumatism	24.7	55.7	32.7	10.1	1.5	1.55	1.44;1.66	< 0.001
Cancer	1.8	54.8	30.1	13.3	1.8	1.52	1.19;1.94	0.001
Diabetes	12.4	53.5	33.4	11.1	2.0	1.62	1.48;1.79	< 0.001
Bronchitis or asthma	8.5	58.6	30.3	10.3	0.8	1.26	1.12;1.41	0.001
Arterial hypertension	43.2	58.0	31.6	9.0	1.4	1.51	1.41;1.61	< 0.001
Heart disease	15.2	53.2	34.3	10.8	1.6	1.65	1.51;1.80	< 0.001
Renal insufficiency	5.0	54.3	33.7	11.0	1.1	1.49	1.29;1.71	< 0.001
Depression	17.7	57.9	30.5	10.1	1.5	1.35	1.24;1.47	< 0.001
Tendonitis	10.0	59.2	30.4	9.4	1.0	1.22	1.10;1.36	< 0.001
Age group (years)								
18 to 34	16.0	73.7	21.6	3.8	1.0	1.00	–	–
35 to 49	33.5	65.2	27.0	6.6	1.2	1.50	1.36;1.66	< 0.001
50 to 65	50.5	58.7	31.4	8.5	1.4	1.98	1.80;2.18	< 0.001
Sex								
Male	25.8	61.6	29.2	7.5	1.7	1.11	1.03;1.19	0.005
Color/Race								
White	45.5	64.0	27.5	7.4	1.1	0.95	0.89;1.02	0.149

^a^ *Per capita* family income in BRL (1st tertile: ≤ 276; 2^nd^ tertile: 277-507; 3^rd^ tertile: > 507).

Half of the participants assessed their health status as moderate and approximately 1/5 of the specimen had health insurance membership and had been hospitalized in the last 12 months. Among the self-referred chronic diseases, back problems were the most frequent, followed by arterial systemic hypertension, arthritis/rheumatism, depression, heart diseases, and diabetes mellitus.

Functional disabilities were self-referred by 36.7% (95%CI 35.4;38.0) of interviewed (Table 2).

**Tabela 2:** Prevalência de incapacidade funcional. Brasil, 2015.

Functional disability*	Prevalence %	95%CI
With no difficulty	63.3	62.5;64.0
With little difficulty	28.3	27.6;29.1
With great difficulty	7.1	6.7;7.5
Not able to do it	1.3	1.1;1.4

* Measured by the difficulty of walking for about 100 m. Prevalence of functional disability. 28.3 + 7.1 + 1.3 = 36.7%

Approximately half of the interviewed who reported presenting functional disabilities had up to three years of study, were economically inactive, assessed their health condition as bad, were hospitalized in the last 12 months, reported presenting some chronic diseases, and had between 50 to 65 years.

By the ordinal logistic regression presented in Table 3 regardless of the scenario adopted, the following variables showed associations with functional disabilities: to reside in urban areas, have lower levels of education and *per capita* household incomes, be economically inactive, have assessed their health condition as bad, have been hospitalized in the last 12 months, present some chronic diseases (arthritis/rheumatism, diabetes mellitus, arterial systemic hypertension, and heart diseases), be with age superior to 34 years and be male. The sensitivity analysis did not change the results.

**Table 3:** Ordinal logistics explanatory models of functional disability of adults. Brazil, 2015.

Variáveis	Scenario 1a			Scenario 2			Scenario 3		

	OR	95%CI	p	OR	95%CI	p	OR	95%CI	p
Block 1^b^									

Health insurance membership									
Yes	0.76	0.70;0.83	< 0.001	0.76	0.70;0.83	< 0.001	0.76	0.70;0.83	< 0.001
Family arrangement									
Living alone	1.09	0.96;1.24	0.172	0.81	0.64;1.02	0.067	0.52	0.27;0.99	0.047
Region of residence									
North	1	–	–	1	–	–	1	–	–
Northeast	0.94	0.85;1.05	0.262	1.05	0.89;1.23	0.562	1.63	1.08;2.46	0.020
Southeast	1.15	1.03;1.28	0.010	1.33	1.14;1.56	< 0.001	1.97	1.32;2.92	0.001
South	1.19	1.06;1.33	0.004	1.19	1.06;1.33	0.004	1.19	1.06;1.33	0.004
Midwest	1.06	0.93;1.21	0.356	1.28	1.05;1.57	0.014	2.53	1.59;4.02	< 0.001
State of domicile									
Urban	1.23	1.12;1.34	0.000	1.23	1.12;1.34	< 0.001	1.23	1.12;1.34	< 0.001

Block 2									

Education level (years)									
≥ 12	1	–	–	1	–	–	1	–	–
8 to 11	1.07	0.95;1.20	0.261	1.31	1.10;1.56	0.002	1.50	1.03;2.18	0.035
4 to 7	1.23	1.11;1.36	< 0.001	1.23	1.11;1.36	< 0.001	1.23	1.11;1.36	< 0.001
0 to 3	1.37	1.23;1.53	< 0.001	1.37	1.23;1.53	< 0.001	1.37	1.23;1.53	< 0.001
*Per capita* household income									
3^rd^ (> 507)	1	–	–	1	–	–	1	–	–
2^nd^ (277-507)	1.01	0.92;1.10	0.903	1.01	0.92;1.10	0.903	1.01	0.92;1.10	0.903
1^st ^(≤ 276)	0.88	0.81;0.97	0.006	0.88	0.81;0.97	0.006	0.88	0.81;0.97	0.006
Economic activity									
Inactive	1.51	1.41;1.61	< 0.001	1.77	1.57;1.99	< 0.001	2.21	1.65;2.96	< 0.001
Self-perception of health									
Good	1	–	–	1	–	–	1	–	–
Moderate	1.47	1.36;1.60	< 0.001	1.47	1.26;1.73	< 0.001	0.93	0.66;1.32	0.699
Bad	2.51	2.27;2.77	< 0.001	3.11	2.63;3.68	< 0.001	1.46	1.00;2.13	0.047
Hospitalization									
In the last 12 months	1.51	1.38;1.65	< 0.001	1.83	1.60;2.10	< 0.001	2.35	1.73;3.20	< 0.001

Block 3									

Chronic diseases									
Back problems	1.02	0.95;1.09	0.598	1.02	0.95;1.09	0.598	1.02	0.95;1.09	0.598
Arthritis/Rheumatism	1.24	1.15;1.34	< 0.001	1.24	1.15;1.34	< 0.001	1.24	1.15;1.34	< 0.001
Cancer	1.19	0.93;1.52	0.159	1.19	0.93;1.52	0.159	1.19	0.93;1.52	0.159
Diabetes	1.16	1.05;1.29	0.004	1.16	1.05;1.29	0.004	1.16	1.05;1.29	0.004
Bronchitis/Asthma	1.11	0.95;1.25	0.097	1.17	0.97;1.41	0.098	0.50	0.27;0.94	0.033
Hypertension	1.10	1.02;1.18	0.010	1.10	1.02;1.18	0.010	1.10	1.02;1.18	0.010
Heart disease	1.13	1.03;1.24	0.009	1.13	1.03;1.24	0.009	1.13	1.03;1.24	0.009
Renal insufficiency	1.11	0.96;1.28	0.171	1.11	0.96;1.28	0.171	1.11	0.96;1.28	0.171
Depression	1.04	0.95;1.14	0.400	1.04	0.95;1.14	0.400	1.04	0.95;1.14	0.400
Tendonitis	1.05	0.94;1.18	0.388	1.05	0.94;1.18	0.388	1.05	0.94;1.18	0.388
Age group (years)									
18 to 34	1	–	–	1	–	–	1	–	–
35 to 49	1.30	1.17;1.45	< 0.001	1.30	1.17;1.45	< 0.001	1.30	1.17;1.45	< 0.001
50 to 65	1.38	1.24;1.54	< 0.001	1.38	1.24;1.54	< 0.001	1.38	1.24;1.54	< 0.001
Sex									
Male	1.17	1.09;1.27	< 0.001	1.17	1.09;1.27	< 0.001	1.17	1.09;1.27	< 0.001
Color/Race									
White	0.96	0.89;1.03	0.241	1.07	0.95;1.20	0.276	0.84	0.63;1.12	0.222

^a^ Scenarios correspond to the following multivariate models: Scenario 1 shows no difficulty *versus* (with little difficulty + with great difficulty + not able to do it); Scenario 2 (with no difficulty + with little difficulty) *versus* (with great difficulty + not able to do it); Scenario 3 (with no difficulty + with little difficulty + with great difficulty) *versus *not able to do it.

^b^ Blocks represent the defined hierarchy for the analysis: Block 1 was adjusted for covariates of the same level; Block 2 was adjusted for covariates of the same level and also by the variables health plan membership and state of domicile; Block 3 was adjusted for covariates of the same level and also by the variables health plan membership, status of residence, education level, household income, economic activity, self-perception of health, and hospitalization.

## DISCUSSION

Four of every 10 adults are affected by functional disabilities. Results of the multivariate model indicate some variables of proximal, intermediate, and distal components were statistically associated with functional disabilities.

There was little variation in the prevalence of functional disabilities in surveys conducted in the Country. The PNAD indicated 25.0% in 1998 and 22.7% in 2003.^16^ Estimates of the World Health Survey (2002 to 2004) pointed out a ratio of 16.8% of functional disabilities in Brazil.^14^ This research still indicated that the frequency of such disabilities in the world is estimated at 15.6%, ranging from a minimum of 4.3% in Ireland and Norway and 35.9% in Swaziland, in South Africa. The National Health Interview Survey (2001 to 2005) has shown that 21.0% of North Americans showed difficulty to walk.^d^ These variations may cause differences in the age of recruitment and in the instruments used during assessment.

In the present study, functional disabilities were measured using the physical mobility variable “difficulty to walk for about 100 meters”, considered as an indicator of moderate functional disability.^12^ The variables “basic activity of daily live” and “difficult to eat, to take a shower, or to go to the bathroom” measure an advanced stage of the disability, not very useful when we think about prevention and intervention. While we point out “difficulty to walk 1 km” as a measurement of active aging and not as an indicator of disability in physical mobility.^19^

Having health insurance membership was a protective factor to functional disability. We presumed individuals affiliated to a plan more often seek these services and have greater adherence to treatments, contributing to the prevention and the improvement of functional capacities.

To reside in urban areas is a significantly associated factor to this limitation. National studies have observed this effect.^4,10^ Adults residing in urban areas feature better life conditions, greater availability, and access to preventive services and specialized medical assistance.^11^

The higher the educational level and the adult’s income, the lower the chance of having functional disabilities, which confirms previous findings.^10,15^ Education determines health advantages, because it promotes access to information, lifestyle changes, insertion of healthy habits, and demands for health services. Economically disadvantaged adults seek less for health services and have little access to treatments and medicines.

We related functional disabilities to the individual’s economic activity. A previous study points out that inactive individuals present few difficulties in daily life activities when compared to those who don’t work.^15^

We associated hospitalization with functional disabilities, reinforcing some previous findings.^2,5^ The immobility syndrome observed in the seventh day of hospitalization induces functional limitations.^7^

We related functional declines to arterial systemic hypertension, diabetes mellitus, arthritis or rheumatism, and heart diseases. These findings are consistent with other studies.^6,21^ Arterial systemic hypertension is a risk factor for strokes and consequent disabilities.^17^ The association between diabetes mellitus and functional disabilities is due to multiple factors, since the disease is related to vascular and neuropathic complications that affect functional capacities.^24^ The damaging of patients’ joints by arthritis or rheumatism hinders greater mobility and movement, leading to disabilities.^8^ Individuals with heart diseases present imbalances between supplies and demands for circulatory nutrients and oxygen to skeletal muscles, potentially affecting physical mobility.

The chance of having functional disabilities is greater in men and also increases with age. National data observed this effect.^8,18^ Aging increases the vulnerability, the risk of diseases, and the prevalence of chronic diseases, which lead to functional disabilities. However, exposure to adverse and inadequate conditions during adult life provides premature functional losses.^20^ In addition, men are more exposed to violence and accidents particularly in youth. Prevention programs must guide young people and not just the older ones.

The cross-sectional study has limitations that suggest the cautious interpretation of our results. It is difficult to interpret associations on causal relationships.^9^ Additionally, the survival bias may be underestimating the observed associations. Besides that, the investigation did not address some variables related to lifestyle that, therefore, were not included in this study.

On the other hand, this analysis provides methodological cares that give greater validity to the results found. Sample weights were considered and we opted for a regression model suitable for this kind of analysis.^1^ We excluded *proxy-*respondents to avoid the risk of information bias and we conducted sensitivity analyses to assess and minimize the effect of chance (type 1 error).

Functional disability is common among Brazilian adults. Hospitalization is the most strongly associated factor, followed by economic activity, and chronic diseases. Sex, age, education, and income are also associated. Results indicate specific targets that address the main factors of functional disabilities and contribute to the projection of interventions for the improvement of the well-being and the promotion of quality of life for adults.

## Footnotes

a World Health Organization. International classification of functioning, disability and health. Geneva: World Health Organization; 2001.

b World Health Organization. Relatório mundial sobre a deficiência. São Paulo (SP): Secretaria dos Direitos da Pessoa com Deficiência; 2012.

c Instituto Brasileiro de Geografia e Estatística. Pesquisa Nacional de Amostra por Domicílio - PNAD, 2008 [CD-ROM]. Rio de Janeiro (RJ): Instituto Brasileiro de Geografia e Estatística; 2008. 1 CD-ROM.

d U.S. Department of Health and Human Services, Centers for Disease Control and Prevention, National Center for Health Statistics. Disability and health in the United States, 2001-2005. Hyattsville: Department of Health and Human Services; 2008. (DHHS Publication, (PHS) 2008-1035).
