# Supplementary material for: Functional disability of adults in Brazil: prevalence and associated factors
Source: Rev Saude Publica. 2015 Dec 16;49:89. doi: 10.1590/S0034-8910.2015049005945 (PMC4687823; doi:10.1590/S0034-8910.2015049005945)
Supplement: Translation

# Translation: Incapacidade funcional de adultos no Brasil: prevalência e fatores associados

**Authors:** Keitty Regina Cordeiro de Andrade, Marcus Tolentino Silva, Taís Freire Galvão, Maurício Gomes Pereira

**Affiliations:** I Faculdade de Medicina. Universidade de Brasília. Brasília, DF, Brasil; II Faculdade de Medicina. Universidade Federal do Amazonas. Manaus, AM, Brasil; III Hospital Universitário Getúlio Vargas. Universidade Federal do Amazonas. Manaus, AM, Brasil

**Keywords:** Adulto, Limitação da Mobilidade, Fatores de Risco, Estatísticas de Sequelas e Incapacidade, Pessoas com Deficiência

## Abstract

**OBJETIVO:**

Estimar a prevalência e os fatores associados à incapacidade funcional em adultos do Brasil.

**MÉTODOS:**

Foram utilizadas as informações do suplemento saúde da Pesquisa Nacional por Amostra de Domicílios de 2008. A variável dependente foi a incapacidade funcional entre adultos de 18 a 65 anos, mensurada pela dificuldade para caminhar cerca de 100 metros e as variáveis independentes foram filiação a plano de saúde, região de residência, situação de domicílio, nível de escolaridade, renda familiar, atividade econômica, autopercepção de saúde, internação hospitalar, doenças crônicas, faixa etária, sexo e cor. Foram calculados os *odds ratios *(OR) brutos e seus respectivos intervalos de confiança (IC95%) e ajustados para variáveis do estudo por meio de regressão logística ordinal seguindo modelo hierarquizado. Os pesos amostrais foram considerados em todos os cálculos.

**RESULTADOS:**

Foram incluídos 18.745 sujeitos, 74,0% dos quais constituíram mulheres. Mais de um terço dos adultos relataram ter incapacidade funcional. A incapacidade foi significativamente maior entre os homens (OR = 1,17; IC95% 1,09;1,27), pessoas de 35 a 49 anos (OR = 1,30; IC95% 1,17;1,45) e 50 a 65 anos (OR = 1,38; IC95% 1,24;1,54); indivíduos inativos economicamente (OR = 2,21; IC95% 1,65;2,96); adultos que reportaram ter doenças cardiovasculares (OR = 1,13; IC95% 1,03;1,24), diabetes mellitus (OR = 1,16; IC95% 1,05;1,29), hipertensão arterial sistêmica (OR = 1,10; IC95% 1,02;1,18), e artrite/reumatismo (OR = 1,24; IC95% 1,15;1,34); e participantes que estiveram internados nos últimos 12 meses (OR = 2,35; IC95% 1,73;3,2).

**CONCLUSÕES:**

Incapacidade funcional é comum entre os adultos brasileiros. A internação hospitalar é o fator mais fortemente associado, seguida de atividade econômica e doenças crônicas. Sexo, idade, escolaridade e renda também são associados. Os resultados indicam alvos específicos para ações que abordem os principais fatores associados à incapacidade funcional e contribuem na projeção de intervenções para a melhora do bem-estar e promoção da qualidade de vida dos adultos brasileiros.

## INTRODUÇÃO

A incapacidade funcional é a dificuldade ou inabilidade de realizar atividades cotidianas básicas dentro dos padrões normais para o ser humano.^a^ A maior causa dessa limitação é a deficiência física, o que gera impacto na habilidade de desenvolver atividades na sociedade.^3,23^

Segundo a Organização Mundial de Saúde cerca de 10,0% da população dos países desenvolvidos é constituída por pessoas com algum tipo de incapacidade funcional e esse percentual sobe para aproximadamente 15,0% nos países em desenvolvimento.^b^

A incapacidade funcional é comumente aferida por autorrelato.^3^ As atividades de vida diária e mobilidade física são frequentemente utilizadas para sua avaliação e são consideradas importantes indicadores do estado de saúde.^13^

A comunidade científica internacional busca entender os fatores associados a esse tema.^14^ Contudo, em âmbito nacional, são escassos estudos de base populacional sobre a prevalência de incapacidade funcional entre os adultos. Conhecer a distribuição e compreender os fatores que colaboram para o prejuízo funcional pode auxiliar os planejadores de políticas públicas na projeção de intervenções para a melhora do bem-estar e promoção da qualidade de vida dos adultos brasileiros.

O presente estudo teve por finalidade estimar a prevalência e os fatores associados a incapacidade funcional de adultos brasileiros.

## MÉTODOS

Análise desenvolvida com base no suplemento saúde da Pesquisa Nacional por Amostra de Domicílios (PNAD). É um inquérito transversal, realizado pelo Instituto Brasileiro de Geografia e Estatística, que obteve informações de uma amostra probabilística de 150.591 domicílios e 391.868 indivíduos, de 28 de setembro de 2007 a 27 de setembro de 2008.^22^

A PNAD dispõe de um plano amostral complexo, planejado para permitir a representatividade nacional, obtido em três estágios de seleção: (a) unidades primárias – municípios autorrepresentativos, com probabilidade de pertencer à amostra, e não autorrepresentativos, com probabilidade de fazer parte da amostra proporcional da população residente; (b) unidades secundárias – setores censitários, onde a probabilidade de inclusão é proporcional ao número de residências existentes no setor; e (c) unidades terciárias – unidades domiciliares (domicílios particulares e unidades de habitação em domicílios coletivos), investigando as informações referentes a todos os residentes.^c^

Este estudo incluiu adultos entre 18 e 65 anos. Apenas as pessoas que informaram sua própria capacidade funcional foram consideradas na análise, e os *proxy*-respondentes foram excluídos.

O questionário do suplemento saúde da PNAD incluiu sete questões acerca de mobilidade física, com respeito a atividades cotidianas, prática de esportes, subir escadas e caminhada. Quatro respostas ordinais eram possíveis: “não consegue”, “tem grande dificuldade”, “tem pequena dificuldade” ou “não tem dificuldade”.

A variável dependente foi a incapacidade funcional, mensurada utilizando a variável de mobilidade física “dificuldade para caminhar cerca de 100 metros”. As variáveis independentes foram determinadas por blocos com componentes distais a proximais (Figura), a fim de evitar subestimação dos efeitos das variáveis distais:

**Figura:** Esquema gráfico do modelo hierárquico empregado na análise ajustada. Brasil, 2015.

Bloco 1, componentes distais: filiação a plano de saúde (sim; não), arranjo familiar (mora sozinho; acompanhado), região de residência (Norte; Nordeste; Sudeste; Sul; Centro-Oeste) e situação de domicílio (rural; urbana).

Bloco 2, componentes intermediários: nível de escolaridade (zero a três anos; quatro a sete anos; oito a 11 anos; e 12 ou mais anos de estudo), renda familiar *per capita *em tercil (3°, > R$507,00; 2°, R$277,00 a R$507,00; e 1°, até R$276,00), atividade econômica (ativo; inativo), autopercepção de saúde (boa; regular; ruim) e internação hospitalar (últimos 12 meses).

Bloco 3, componentes proximais: doenças crônicas (problemas na coluna; artrite/reumatismo; câncer, diabetes mellitus; bronquite ou asma; hipertensão arterial sistêmica; doença cardíaca; insuficiência renal; depressão, tendinite); faixa etária (em anos), sexo (masculino; feminino) e cor (branca; não branca).

Foi obtida a estatística descritiva das variáveis, estratificadas pela incapacidade funcional. Procedeu-se o cálculo da prevalência autorreferida na população total com respectivo intervalo de 95% de confiança (IC95%). Empregou-se a análise bivariada para identificar os fatores associados à incapacidade funcional, adotando como medida de efeito o *odds ratio* (OR).

Os OR ajustados foram calculados utilizando modelo de regressão logística ordinal^1^ que partiu de três cenários: (i) não tem dificuldade *versus* (tem pequena + tem grande + não consegue); (ii) (não tem dificuldade + tem pequena) *versus* (tem grande + não consegue); e (iii) (não tem dificuldade + tem pequena + tem grande) *versus* não consegue. Tal cuidado foi necessário devido à falta de linearidade matemática entre as categorias do desfecho em análise.

A análise multivariada foi hierarquizada pelos blocos previamente definidos. Para cada bloco de análise, foram mantidas no modelo as variáveis com valores de p < 0,10. As variáveis foram ajustadas pelas covariáveis do mesmo nível e pelas variáveis significantes do nível anterior. Utilizou-se a técnica *jackknife *para a análise de sensibilidade, obtendo simulações estratificadas por Unidade da Federação.

As análises foram realizadas utilizando o *software* estatístico Stata versão 10.1. Em todos os cálculos foram considerados os pesos amostrais da PNAD 2008.

A PNAD foi aprovada pela Comissão Nacional de Ética em Pesquisa.

## RESULTADOS

No total, 18.745 entrevistas foram incluídas no estudo. A população era predominantemente feminina, e a maior parte dos adultos tinha entre 50 e 65 anos, morava acompanhada, residia em área urbana, referia sua cor como não branca, tinha até sete anos de estudo, pertencia ao menor tercil de renda e era inativo economicamente (Tabela 1).

**Tabela 1:** Distribuição das características dos adultos e suas associações com a incapacidade funcional. Brasil, 2015. (N = 18.745)

Variáveis	Adultos (%)	Incapacidade funcional (%)				Análise bivariada		

		Não tem dificuldade	Tem pequena dificuldade	Tem grande dificuldade	Não consegue	OR	IC95%	p
Filiação a plano de saúde								
Sim	20,2	67,2	24,9	6,6	1,3	0,81	0,75;0,89	< 0,001
Arranjo familiar								
Mora sozinho	7,3	60,8	32,0	6,4	0,7	1,08	0,98;1,21	0,203
Região de residência								
Norte	8,3	64,6	28,9	6,0	0,6	1,00	–	–
Nordeste	28,0	66,2	26,4	6,3	1,2	0,95	0,86;1,06	0,371
Sudeste	40,0	61,7	29,1	7,8	1,4	1,16	1,05;1,29	0,005
Sul	16,7	61,4	30,0	7,6	1,0	1,16	1,04;1,30	0,010
Centro-Oeste	7,0	63,4	27,6	7,2	1,8	1,09	0,96;1,24	0,181
Situação de domicílio								
Urbana	82,4	62,5	29,0	7,3	1,3	1,22	1,12;1,33	< 0,001
Nível de escolaridade (anos)								
≥ 12	21,0	70,0	24,2	4,6	1,2	1,00	–	–
8 a 11	16,6	66,4	25,3	6,9	1,4	1,21	1,08;1,35	0,001
4 a 7	33,9	61,7	29,9	7,4	1,0	1,45	1,32;1,59	< 0,001
0 a 3	28,4	58,4	31,4	8,8	1,5	1,68	1,53;1,85	< 0,001
Renda familiar* per capita* (tercil)^a^								
3° (> 507)	30,0	65,3	27,0	6,5	1,2	1,00	–	–
2° (277-507)	28,6	61,2	29,7	7,6	1,5	1,19	1,09;1,30	< 0,001
1° (≤ 276)	41,4	63,2	28,4	7,3	1,1	1,09	1,01;1,18	0,027
Atividade econômica								
Inativo	43,4	56,7	32,0	9,5	1,8	1,68	1,58;1,79	< 0,001
Autopercepção de saúde								
Boa	31,0	73,0	22,2	3,8	1,1	1,00	–	–
Regular	50,3	62,9	29,4	6,6	1,1	1,58	1,47;1,71	< 0,001
Ruim	18,7	48,2	35,7	14,2	2,0	3,03	2,76;3,34	< 0,001
Internação hospitalar								
Últimos 12 meses	16,0	53,1	32,8	11,6	2,5	1,73	1,59;1,88	< 0,001
Doenças crônicas								
Problemas na coluna	46,0	60,7	29,9	8,4	1,2	1,24	1,16;1,32	< 0,001
Artrite ou reumatismo	24,7	55,7	32,7	10,1	1,5	1,55	1,44;1,66	< 0,001
Câncer	1,8	54,8	30,1	13,3	1,8	1,52	1,19;1,94	0,001
Diabetes	12,4	53,5	33,4	11,1	2,0	1,62	1,48;1,79	< 0,001
Bronquite ou asma	8,5	58,6	30,3	10,3	0,8	1,26	1,12;1,41	0,001
Hipertensão arterial	43,2	58,0	31,6	9,0	1,4	1,51	1,41;1,61	< 0,001
Doença cardíaca	15,2	53,2	34,3	10,8	1,6	1,65	1,51;1,80	< 0,001
Insuficiência renal	5,0	54,3	33,7	11,0	1,1	1,49	1,29;1,71	< 0,001
Depressão	17,7	57,9	30,5	10,1	1,5	1,35	1,24;1,47	< 0,001
Tendinite	10,0	59,2	30,4	9,4	1,0	1,22	1,10;1,36	< 0,001
Faixa etária (anos)								
18 a 34	16,0	73,7	21,6	3,8	1,0	1,00	–	–
35 a 49	33,5	65,2	27,0	6,6	1,2	1,50	1,36;1,66	< 0,001
50 a 65	50,5	58,7	31,4	8,5	1,4	1,98	1,80;2,18	< 0,001
Sexo								
Masculino	25,8	61,6	29,2	7,5	1,7	1,11	1,03;1,19	0,005
Cor/Raça								
Branca	45,5	64,0	27,5	7,4	1,1	0,95	0,89;1,02	0,149

^a^ Renda familiar *per capita *em R$ (1º tercil: ≤ 276; 2° tercil: 277-507; 3° tercil: > 507).

Metade dos participantes avaliou seu estado de saúde como regular e aproximadamente 1/5 da amostra possuía plano e esteve internada nos últimos 12 meses. Dentre as doenças crônicas autorreferidas, problema na coluna foi a mais frequente, seguida por hipertensão arterial sistêmica, artrite/reumatismo, depressão, doença cardíaca e diabetes mellitus.

Incapacidade funcional foi autorreferida por 36,7% (IC95% 35,4;38,0) dos entrevistados (Tabela 2).

**Tabela 2:** Prevalência de incapacidade funcional. Brasil, 2015.

Incapacidade funcional*	Prevalência (%)	IC95%
Não tem dificuldade	63,3	62,5;64,0
Tem pequena dificuldade	28,3	27,6;29,1
Tem grande dificuldade	7,1	6,7;7,5
Não consegue	1,3	1,1;1,4

* Medida pela dificuldade para andar cerca de 100 m. Prevalência de incapacidade: 28,3 + 7,1 + 1,3 = 36,7%.

Aproximadamente metade dos entrevistados que relataram ter incapacidade funcional tinha até três anos de estudo, era inativo economicamente, avaliou seu estado de saúde como ruim, esteve internado nos últimos 12 meses, relatou ter alguma doença crônica e tinha entre 50 a 65 anos.

Pela regressão logística ordinal, apresentada na Tabela 3, independentemente do cenário adotado, as seguintes variáveis apresentaram associação com a incapacidade funcional: residir em área urbana, dispor de menor nível de escolaridade e de renda familiar *per capita*, estar inativo economicamente, ter autopercepção de saúde ruim, ter sido hospitalizado nos últimos 12 meses, ter algumas doenças crônicas (artrite/reumatismo, diabetes mellitus, hipertensão arterial sistêmica e doença cardíaca), estar com idade superior a 34 anos e ser homem. A análise de sensibilidade não alterou os resultados encontrados.

**Tabela 3:** Modelos logísticos ordinais explicativos da incapacidade funcional de adultos. Brasil, 2015.

Variáveis	Cenário 1^a^			Cenário 2			Cenário 3		

	OR	IC95%	p	OR	IC95%	p	OR	IC95%	p
Bloco 1^b^									

Filiação a plano de saúde									
Sim	0,76	0,70;0,83	< 0,001	0,76	0,70;0,83	< 0,001	0,76	0,70;0,83	< 0,001
Arranjo familiar									
Mora sozinho	1,09	0,96;1,24	0,172	0,81	0,64;1,02	0,067	0,52	0,27;0,99	0,047
Região de residência									
Norte	1	–	–	1	–	–	1	–	–
Nordeste	0,94	0,85;1,05	0,262	1,05	0,89;1,23	0,562	1,63	1,08;2,46	0,020
Sudeste	1,15	1,03;1,28	0,010	1,33	1,14;1,56	< 0,001	1,97	1,32;2,92	0,001
Sul	1,19	1,06;1,33	0,004	1,19	1,06;1,33	0,004	1,19	1,06;1,33	0,004
Centro-Oeste	1,06	0,93;1,21	0,356	1,28	1,05;1,57	0,014	2,53	1,59;4,02	< 0,001
Situação de domicílio									
Urbana	1,23	1,12;1,34	0,000	1,23	1,12;1,34	< 0,001	1,23	1,12;1,34	< 0,001

Bloco 2									

Nível de escolaridade (anos)									
≥ 12	1	–	–	1	–	–	1	–	–
8 a 11	1,07	0,95;1,20	0,261	1,31	1,10;1,56	0,002	1,50	1,03;2,18	0,035
4 a 7	1,23	1,11;1,36	< 0,001	1,23	1,11;1,36	< 0,001	1,23	1,11;1,36	< 0,001
0 a 3	1,37	1,23;1,53	< 0,001	1,37	1,23;1,53	< 0,001	1,37	1,23;1,53	< 0,001
Renda familiar *per capita *(tercil)									
3° (> 507)	1	–	–	1	–	–	1	–	–
2° (277-507)	1,01	0,92;1,10	0,903	1,01	0,92;1,10	0,903	1,01	0,92;1,10	0,903
1° (≤ 276)	0,88	0,81;0,97	0,006	0,88	0,81;0,97	0,006	0,88	0,81;0,97	0,006
Atividade econômica									
Inativo	1,51	1,41;1,61	< 0,001	1,77	1,57;1,99	< 0,001	2,21	1,65;2,96	< 0,001
Autopercepção de saúde									
Boa	1	–	–	1	–	–	1	–	–
Regular	1,47	1,36;1,60	< 0,001	1,47	1,26;1,73	< 0,001	0,93	0,66;1,32	0,699
Ruim	2,51	2,27;2,77	< 0,001	3,11	2,63;3,68	< 0,001	1,46	1,00;2,13	0,047
Internação hospitalar									
Últimos 12 meses	1,51	1,38;1,65	< 0,001	1,83	1,60;2,10	< 0,001	2,35	1,73;3,20	< 0,001

Bloco 3									

Doenças crônicas									
Problemas na coluna	1,02	0,95;1,09	0,598	1,02	0,95;1,09	0,598	1,02	0,95;1,09	0,598
Artrite/Reumatismo	1,24	1,15;1,34	< 0,001	1,24	1,15;1,34	< 0,001	1,24	1,15;1,34	< 0,001
Câncer	1,19	0,93;1,52	0,159	1,19	0,93;1,52	0,159	1,19	0,93;1,52	0,159
Diabetes	1,16	1,05;1,29	0,004	1,16	1,05;1,29	0,004	1,16	1,05;1,29	0,004
Bronquite/Asma	1,11	0,95;1,25	0,097	1,17	0,97;1,41	0,098	0,50	0,27;0,94	0,033
Hipertensão	1,10	1,02;1,18	0,010	1,10	1,02;1,18	0,010	1,10	1,02;1,18	0,010
Doença cardíaca	1,13	1,03;1,24	0,009	1,13	1,03;1,24	0,009	1,13	1,03;1,24	0,009
Insuficiência renal	1,11	0,96;1,28	0,171	1,11	0,96;1,28	0,171	1,11	0,96;1,28	0,171
Depressão	1,04	0,95;1,14	0,400	1,04	0,95;1,14	0,400	1,04	0,95;1,14	0,400
Tendinite	1,05	0,94;1,18	0,388	1,05	0,94;1,18	0,388	1,05	0,94;1,18	0,388
Faixa etária (anos)									
18 a 34	1	–	–	1	–	–	1	–	–
35 a 49	1,30	1,17;1,45	< 0,001	1,30	1,17;1,45	< 0,001	1,30	1,17;1,45	< 0,001
50 a 65	1,38	1,24;1,54	< 0,001	1,38	1,24;1,54	< 0,001	1,38	1,24;1,54	< 0,001
Sexo									
Masculino	1,17	1,09;1,27	< 0,001	1,17	1,09;1,27	< 0,001	1,17	1,09;1,27	< 0,001
Cor/Raça									
Branca	0,96	0,89;1,03	0,241	1,07	0,95;1,20	0,276	0,84	0,63;1,12	0,222

^a^ Cenários correspondem aos modelos multivariados: Cenário 1, não tem dificuldade x (tem pequena dificuldade + tem grande dificuldade + não consegue); Cenário 2, (não tem dificuldade + tem pequena dificuldade) x (tem grande dificuldade + não consegue); Cenário 3, (não tem dificuldade + tem pequena dificuldade + tem grande dificuldade) x não consegue.

^b^ Blocos representam a hierarquia definida para a análise: Bloco 1, ajustado pelas covariáveis do mesmo nível; Bloco 2, ajustado pelas covariáveis do mesmo nível e também pelas variáveis filiação a plano de saúde e situação de domicílio; Bloco 3, ajustado pelas covariáveis do mesmo nível e também pelas variáveis filiação a plano de saúde, situação de domicílio, nível de escolaridade, renda familiar, atividade econômica, autopercepção de saúde e internação hospitalar.

## DISCUSSÃO

A cada 10 adultos brasileiros, quatro são afetados por incapacidade funcional. Os resultados do modelo multivariado indicam que algumas variáveis dos componentes proximais, intermediários e distais foram estatisticamente associadas à limitação funcional.

Houve pequena variação da prevalência de incapacidade funcional nos inquéritos realizados no País. A PNAD indicou 25,0% em 1998 e 22,7% em 2003.^16^ Estimativa da Pesquisa Mundial de Saúde (2002 a 2004) apontou proporção de 16,8% dessa limitação funcional no Brasil.^14^ Essa pesquisa indicou ainda que a frequência no mundo é estimada em 15,6%, variando de um mínimo de 4,3% na Irlanda e Noruega e 35,9% na Suazilândia na África do Sul. O *Nacional Health Interview Survey *(2001 a 2005) mostrou que 21,0% dos americanos tinham dificuldade para caminhar.^d^ Tais variações podem refletir diferenças na idade de recrutamento e nos instrumentos usados na aferição.

No presente estudo, incapacidade funcional foi mensurada utilizando a variável de mobilidade física “dificuldade para caminhar cerca de 100 metros”, considerada um indicador de incapacidade funcional moderado.^12^ As variáveis “atividade básica da vida diária” e “dificuldade para alimentar-se, tomar banho ou ir ao banheiro” mensuram um estágio avançado da incapacidade, pouco útil quando se pensa em prevenção e intervenção. Já “dificuldade para caminhar 1 km” é apontada como uma mensuração de envelhecimento ativo e não um indicador de incapacidade em mobilidade física.^19^

Ter plano de saúde foi fator protetor à incapacidade funcional. É presumível que indivíduos filiados a um plano busquem mais frequentemente os serviços e tenham maior adesão aos tratamentos, colaborando para a prevenção e melhora da capacidade funcional.

Residir em área urbana é fator associado significativamente a essa limitação. Estudos nacionais observaram esse efeito.^4,10^ Adultos que residem em áreas urbanas apresentam melhores condições de vida, maior disponibilidade e acesso aos serviços preventivos e assistência médica especializada.^11^

Quanto maior o nível educacional e a renda do adulto, menor a chance de ter incapacidade funcional, o que confirma achados prévios.^10,15^ A educação determina vantagens para a saúde, pois favorece o acesso a informações, mudança do estilo de vida, inserção de hábitos saudáveis e procura por serviços de saúde. Os adultos hipossuficientes procuram menos os serviços de saúde e possuem pouco acesso aos tratamentos e medicamentos.

A incapacidade funcional relacionou-se com a atividade econômica do indivíduo. Estudo prévio aponta que indivíduos inativos apresentam poucas dificuldades com as atividades de vida diária quando comparados àqueles que não trabalham.^15^

A internação hospitalar associou-se com a incapacidade funcional, reforçando achados prévios.^2,5^ A síndrome do imobilismo observada no sétimo dia de internação hospitalar induz limitações funcionais.^7^

Declínio funcional associou-se com hipertensão arterial sistêmica, diabetes mellitus, artrite ou reumatismo, e doença cardíaca. Tais achados são consistentes com outros estudos.^6,21^ A hipertensão arterial sistêmica é fator de risco para o acidente vascular encefálico e consequente incapacidade.^17^ A associação entre diabetes mellitus e incapacidade funcional é devida a múltiplos fatores, pois a doença está relacionada, sobretudo, a complicações vasculares e neuropáticas que afetam a capacidade funcional.^24^ O comprometimento das articulações de pacientes com artrite ou reumatismo impedem maior mobilidade e movimento, induzindo a incapacidade.^8^ Indivíduos com doença cardíaca apresentam desequilíbrio entre o suprimento e a demanda circulatória de nutrientes e oxigênio para a musculatura esquelética, afetando potencialmente a mobilidade física.

A chance de ter incapacidade funcional é maior entre os homens e aumenta com a idade. Dados nacionais observaram esse efeito.^8,18^ O envelhecimento aumenta a vulnerabilidade, os riscos de agravos e a prevalência de doenças crônicas, que induzem à incapacidade. Contudo, a exposição a condições adversas e inadequadas durante a vida adulta propicia prejuízos funcionais precocemente.^20^ Além disso, os homens são mais expostos à violência e acidentes em particular durante a juventude. Programas de prevenção devem orientar os jovens e não apenas a população idosa.

O estudo transversal tem limitações que sugerem interpretação cautelosa dos nossos resultados. É difícil interpretar associações em termos de relação causal.^9^ Adicionalmente, o viés de sobrevivência pode estar subestimando as associações observadas. Afora disso, o inquérito não abordou algumas variáveis relacionadas ao estilo de vida e, portanto, não foram incluídas no presente estudo.

Por outro lado, esta análise dispõe de cuidados metodológicos que conferem maior validade aos resultados encontrados. Os pesos amostrais foram ponderados e optou-se por um modelo de regressão apropriado para esse tipo de análise.^1^ Foram excluídos os *proxy*-respondentes para evitar o risco de viés de informação e realizaram-se análises de sensibilidade para avaliar e minimizar o efeito do acaso (erro tipo 1).

Incapacidade funcional é comum entre os adultos brasileiros. A internação hospitalar é o fator mais fortemente associado, seguida de atividade econômica e doenças crônicas. Sexo, idade, escolaridade e renda também são associados. Os resultados indicam alvos específicos para ações que abordem os principais fatores associados à incapacidade funcional, e contribuem na projeção de intervenções para a melhora do bem-estar e promoção da qualidade de vida dos adultos brasileiros.

## Footnotes

a World Health Organization. International classification of functioning, disability and health. Geneva: World Health Organization; 2001.

b World Health Organization. Relatório mundial sobre a deficiência. São Paulo: Secretaria dos Direitos da Pessoa com Deficiência; 2012.

c Instituto Brasileiro de Geografia e Estatística. Pesquisa Nacional de Amostra por Domicílio - PNAD, 2008 [CD-ROM]. Rio de Janeiro: Instituto Brasileiro de Geografia e Estatística; 2008. 1 CD-ROM.

d U.S. Department of Health and Human Services, Centers for Disease Control and Prevention, National Center for Health Statistics. Disability and health in the United States, 2001-2005. Hyattsville: Department of Health and Human Services; 2008. (DHHS Publication, (PHS) 2008-1035).
